# A multicenter study by the Brazilian Society of Dermatology on a new standard patch test series^؆^

**DOI:** 10.1016/j.abd.2026.501327

**Published:** 2026-03-27

**Authors:** Renan Rangel Bonamigo, Vanessa Barreto Rocha, Vitor Manoel Silva dos Reis, Dulcilea Ferraz Rodrigues, Rosana Lazzarini, Mario Cezar Pires, Renato Shintani Hikawa, Ana Elisa Kiszewski, Andrea Fernandes Eloy da Costa França, Renata Heck, Maria Antonieta Rios Scherrer, Luciana Paula Samorano, Isabella Campos Machado Cordeiro, Ida Alzira Duarte, Mariana de Figueiredo Silva Hafner, Renata Marli Gonçalves Pires, Lilith Sodré Eller, Juliana Barros Rodrigues, Daniela Benzano Bumaguin, Ruppert Ludwig Hahnstadt

**Affiliations:** a Service of Dermatology, Hospital de Clínicas de Porto Alegre, Universidade Federal do Rio Grande do Sul, Porto Alegre, RS, Brazil; b Department of Internal Medicine, Faculdade de Medicina, Universidade Federal de Minas Gerais, Belo Horizonte, MG, Brazil; c Department of Dermatology, Faculdade de Medicina, Universidade de São Paulo, São Paulo, SP, Brazil; d Service of Dermatology, Santa Casa de Belo Horizonte, Belo Horizonte, MG, Brazil; e Faculdade de Ciências Médicas, Santa Casa de São Paulo, São Paulo, SP, Brazil; f Complexo Hospitalar Padre Bento de Guarulhos, Guarulhos, SP, Brazil; g Hospital do Servidor Público do Estado de São Paulo, São Paulo, SP, Brazil; h Department of Dermatology, Universidade Federal de São Paulo, São Paulo, SP, Brazil; i Service of Dermatology, Department of Internal Medicine, Santa Casa de Porto Alegre, Porto Alegre, RS, Brazil; j Faculdade de Ciências Médicas Universidade Estadual de Campinas, Campinas, SP, Brazil; k Service of Dermatology, Hospital das Clínicas, Universidade Federal de Minas Gerais, Belo Horizonte, MG, Brazil; l Dermatology Clinic, Santa Casa de São Paulo, São Paulo, SP, Brazil; m Service of Dermatology, Santa Casa de Porto Alegre, Porto Alegre, RS, Brazil; n Universidade Federal do Rio Grande do Sul, Porto Alegre, RS, Brazil; o Immunotech Laboratories, FDA-Allergenics, Rio de Janeiro, RJ, Brazil

**Keywords:** Dermatitis, contact, Eczema, Epidemiology, Patch test

## Abstract

**Background:**

The prevalence of allergic contact dermatitis (ACD) differs across various populations, and the sensitization rate can change constantly as new products are introduced to the market. The current Brazilian standard series of patch tests has been used for over two decades. Therefore, the development of a new Brazilian standard series is important to ensure that contact tests remain a reliable diagnostic tool.

**Methods:**

A Brazilian multicenter, cross-sectional, prospective study was conducted using a modified series of patch tests between 2023 and 2025. The factors under study were the substances used in the contact tests, and the outcomes were the frequencies of positivity and relevance. Demographic and clinical variables of the participants, who had contact dermatitis without a defined etiology prior to the tests, were evaluated. The required sample size was defined as 523 patients, estimating the prevalence of positivity with a 1.8% confidence interval. Wald's test was considered a 95% confidence level and a 1% expected prevalence of positivity.

**Results:**

A total of 567 participants were included, 77.3% female, with a mean age of 53.3 years, and most belonged to phototypes II, III, and IV. Cleaning services were the most frequent occupational activity, and 43.3% of patients had a personal history of atopy. The overall reactivity of the tests was 69.3%, with 70.2% relevance. Nickel sulfate was the most frequently reactive allergen (28.9%), and 29 substances reached at least 1% positivity.

**Limitations:**

Lack of representatives from all regions of Brazil and absence of prospective follow-up.

**Conclusion:**

The authors propose a new Brazilian standard series of patch tests, with 40 substances, considering the 29 that achieved 1% positivity and another 11 with clinical importance and supported by current literature as emerging and current allergens.

## Introduction

Allergic contact dermatitis (ACD) is a disease with high frequency, clinical and occupational importance, and can affect any sex and age group.^1^

The disease is caused by allergens that sensitize the individual through a delayed-type hypersensitivity reaction (type IV) and, in its constitution, has multifactorial risk factors, such as certain professions, the state of the skin barrier, and the frequency and intensity of exposure to substances with greater allergenic potential.^1^, ^2^

It is estimated that there are more than 4,000 substances related to ACD; of these, 300 to 400 substances are relevant in clinical practice and are components of patch test series in different countries worldwide.^1^, ^2^, ^3^

The prevalence of ACD differs in various populations, being influenced by the profile of each region. Furthermore, the sensitization rate can change constantly as new products are introduced and others are withdrawn from the market. Added to this are changes in the behavior and usage preferences of populations, also influenced by consumption trends. Therefore, the presence of and exposure to sensitizers change over time.^3^, ^4^

Patch tests (PT – or epicutaneous tests) are considered the gold standard complementary tests for the etiological diagnosis of ACD.^4^, ^5^, ^6^, ^7^, ^8^ This technique was developed by Jadassohn more than 100 years ago and formally described by Sulzberger and Wise in 1931.^8^ The tests consist of applying substances, in pre-established concentrations, to the patient's skin, in an attempt to reproduce at the test site an inflammatory reaction similar to the patient's dermatosis and thus confirm the CD etiology.^8^

The current Brazilian standard series for identifying the etiologies of contact dermatitis has been used for over two decades and was developed from studies and discussions among experts from the Brazilian Contact Dermatitis Study Group – GBEDC (Table 1).^9^

**Table 1:** Brazilian Standard Series (2000‒2025).

**Substances concentrations and vehicles**
1. Anthraquinone 2% solid petroleum jelly
2. Balsam of Peru 25% solid petroleum jelly
3. PPD mix 0,4% solid petroleum jelly
4. Hydroquinone 1% solid petroleum jelly
5. Potassium dichromate 0,5% solid petroleum jelly
6. Propylene glycol 10% solid petroleum jelly
7. Tertiary p-butylphenol 1% solid petroleum jelly
8. Neomycin 20% solid petroleum jelly
9. Irgasan 1% solid petroleum jelly
10. Kathon CG® 0,5% solid petroleum jelly
11. Cobalt chloride 1% solid petroleum jelly
12. Lanoline 30% solid petroleum jelly
13. Thiuram mix 1% solid petroleum jelly
14. Ethylenediamine 1% solid petroleum jelly
15 Perfume mix 7% solid petroleum jelly
16. Mercapto mix 2% solid petroleum jelly
17. Benzocaine 5% solid petroleum jelly
18. Quaternium 0,5 solid petroleum jelly
19. Quinoline mix 6% solid petroleum jelly
20. Nitrofurazone 1% solid petroleum jelly
21. Paraben mix 15% solid petroleum jelly
22. Epoxy (resin) 1% solid petroleum jelly
23. Timerosal 0,05% solid petroleum jelly
24. Turpentine 10% solid petroleum jelly
25. Carba mix 3% solid petroleum jelly
26. Promethazine 1% solid petroleum jelly
27. Nickel sulfate 5% solid petroleum jelly
28. Colophony 20% solid petroleum jelly
29. Paraphenylenediamine 1% solid petroleum jelly
30. Formaldehyde 1% solid water

In a recent systematic review of the literature covering Brazilian data between 2000 and 2022, using the current patch test series, it was found that nickel sulfate was the most frequently positive allergen, while triclosan, tertiary p-butylphenol, and anthraquinone were the least prevalent. Interestingly, the prevalence of positive tests increased every five years for Kathon CG®, neomycin, and nickel sulfate, and decreased for Quaternium-15 and thimerosal.^10^

The development of a new Brazilian standard series is important to ensure that patch tests remain a reliable diagnostic tool with high accuracy, contributing to the etiological clarification of ACD today. For this purpose, a new and comprehensive study is necessary. The results of the new research should be added to studies published in the literature over the past 20 years.

Thus, the present study aimed to test substances from the current Brazilian standard series, plus others, which were defined as being considered emerging and potentially important. In the end, considering the new data, recent historical statistical aspects, and the discussion among expert authors, a new Brazilian standard series for patch tests is suggested.

## Method

This multicenter study was conducted with the support of the Brazilian Society of Dermatology (SBD, *Sociedade Brasileira de Dermatologia*), under the coordination of the Department of Allergy and Occupational Dermatoses.

The authors developed a cross-sectional and prospective diagnostic intervention study between 2023 and 2025. Under the coordination of the Department of Allergy and Occupational Dermatoses of the Brazilian Society of Dermatology, ten accredited Services participated as research centers (i, ii, iii, iv, v, vi, vii, viii, ix, x). The factors under study were the substances in the PT, and the outcomes were their respective frequencies of positivity in the final reading (96 h).

The minimum sample required for the study was defined as 470 patients, considering estimating the proportion of occurrence of the positivity prevalence with a 1.8% amplitude for the confidence interval. With the addition of 10% for possible losses and refusals, this number reached 523. The calculation using Wald’s method considered a confidence level of 95% and a 1% expected percentage for the prevalence of positivity. These calculations were performed using the PSS Health online tool.

The study was approved by the Ethics and Research Committees of each of the ten centers (Hospital de Clínicas de Porto Alegre da Universidade Federal do Rio Grande do Sul, Hospital das Clínicas da UFMG, Santa Casa de Porto Alegre, Santa Casa de Belo Horizonte, Santa Casa de São Paulo, Hospital das Clínicas da USP, Complexo Hospitalar Padre Bento, Hospital do Servidor Público Estadual de São Paulo, Hospital das Clínicas da Universidade Federal de São Paulo, Universidade Estadual de Campinas, Universidade Federal do Rio Grande do Sul). The coordinating center for the multicenter study was the Dermatology Service of Hospital de Clínicas de Porto Alegre, with approval registered under CAAE number 67648123.7.1001.5327.

Participants were patients with a suspected diagnosis of ACD, treated at the centers described above, and with an indication for patch testing according to the protocols of the Dermatology Services.

Patients aged 18 years or older who understood and agreed to participate in the study by signing the informed consent form were included.

Exclusion criteria were: physical or mental disabilities, pregnant women, patients using systemic corticosteroids in the last 30 days, use of topical corticosteroids at the test site in the last seven days, use of systemic immunosuppressants (methotrexate, cyclosporine, azathioprine, mycophenolate mofetil, immunobiologic drugs, JAK pathway inhibitors and/or others) in the last three months, active dermatosis or other dermatological conditions at the test site, history of anaphylaxis, history of sun exposure in the test area in the 15 days prior to the examination, history of exposure to ultraviolet radiation through phototherapy or booths in the test area in the 30 days prior to the examination.

The independent variables collected and analyzed were sex, age, phototype, occupation, origin, history of atopy, dermatitis topography, and each tested substance; and the dependent variables were the results of the PT readings and the relevance for each substance, performed after 48 and 96 hours.

The material used was a modified Brazilian standard series of patch tests, which included 50 substances (among those existing in the current series and others, defined from consensus meetings among SBD experts). The material (substances, chambers, recording pen, and explanatory media standardizing the testing) was produced and made available by the company FDA Allergenics.

The retainers used were those standardized by the International Contact Dermatitis Study Group – ICDRG (FINN CHAMBERS AQUA® – SmartPractice; Phoenix – AZ, United States of America, USA), with 10 hypoallergenic tape chambers in each retainer.

The 50 substances that constituted the modified series are shown in Table 2. By decision of the authors, Quaternium, nitrofurazone, quinoline, and anthraquinone were not tested from the current series, due to their progressively decreasing positive rates over two decades. Lanolin was replaced by Amerchol® L 101 (as it is an integral part of it), and benzocaine is part of Caine Mix I, therefore, it was replaced by it. In addition, thimerosal is a preservative that is no longer in use, so it was not tested, either.^10^

**Table 2:** Modified standard series under evaluation (2023-2025).

Modified standard series under evaluation (2023-2025)^a^		
Number	Substances and concentrations (%)	Vehicle
1	Potassium Dichromate 0.5	Solid petroleum jelly
2	Balsam of Peru 25	Solid petroleum jelly
3	Ketoconazole 1	Solid petroleum jelly
4	Fragrance mix II	Solid petroleum jelly
	Coumarin 2,5	
	Farnesol 2,5	
	Lyral 2,5	
	Citral 1	
	Citronellal 0,5	
	5-Aldehyde-hexylcinnamaldehyde	
5	Bisphenol A 1 epoxy resin	Solid petroleum jelly
6	Propylene glycol 10	Solid petroleum jelly
7	Tertiary p-butylphenol 1	Solid petroleum jelly
8	Neomycin 20	Solid petroleum jelly
9	Irgasan 1	Solid petroleum jelly
10	PPD mix	Solid petroleum jelly
	N-isopropyl, n-phenyl, Paraphenylenediamine 0.2	
	N-n diphenyl, Paraphenylenediamine 0.2	
11	Imidazolidinyl Urea 2	Solid petroleum jelly
12	Phenoxyethanol 1	Solid petroleum jelly
13	Thiuram Mix	Solid petroleum jelly
	Tetramethylthiuramdisulfide 0.5	
	Tetramethylthiurandisulfide 0.5	
14	Ethylenediamine 1	Solid petroleum jelly
15	Disperse Blue 106 1	Solid petroleum jelly
16	Mercapto Mix	Solid petroleum jelly
	Mercaptobenzothiazole 0.5	
	Dibenzothiazole Disulfide 0.5	
	Morpholinylmercaptobenzothiazole 0.5	
	N-Cyclohexyl 2 Benzothiazol Sulfonamide 0.5	
17	Caine mix I	Solid petroleum jelly
	Benzocaine 5	
	Tetracaine 2.5	
	Dibucaine 2.5	
18	Fragrance Mix I	Solid petroleum jelly
	Cinnamic alcohol 2	
	alpha-amyl cinnamaldehyde 2	
	Eugenol 2	
	Isoeugenol 2	
	Geraniol 2	
	Hydroxycitronellal 2	
	Oak moss absolute 2	
19	Hydroxyethyl Methacrylate 2	Solid petroleum jelly
20	Cobalt Chloride 1	Solid petroleum jelly
21	Paraben mix	Solid petroleum jelly
	Methylparaben 3	
	Ethylparaben 3	
	Propylparaben 3	
	Butylparaben 3	
	Benzylparaben 3	
22	Bronopol 0.5	Solid petroleum jelly
23	DMDM Hydantoin 1	Solid petroleum jelly
24	*Compositae* mix 2.5	Solid petroleum jelly
	*Anthemis nobilis* extract 0.6	
	*Chamomilla recutita* extract 0.6	
	*Achillea millefolium* extract 0.5	
	*Tanacetum vulgare* extract 0.5	
	*Arnica montana* extract 0.25	
	Parthenolide 0.05	
25	Carba mix	Solid petroleum jelly
	Diphenylguanidine 1	
	Zinc dimethyldithiocarbamate 1	
	Zinc diethyldithiocarbamate 1	
26	Colophony 20	Solid petroleum jelly
27	Nickel Sulfate 5	Solid petroleum jelly
28	TOSYLAMIDE 10	Solid petroleum jelly
29	Paraphenylenediamine 1	Solid petroleum jelly
30	Limonene Hydroperoxide 0.3	Solid petroleum jelly
31	*Compositae* mix 5	Solid petroleum jelly
	*Anthemis nobilis* extract 1.2	
	*Chamomilla recutita* extract 1.2	
	*Achillea millefolium* extract 1	
	*Tanacetum vulgare* extract 1	
	*Arnica montana* extract 0.5	
	Parthenolide 0,1	
32	N-Isopropyl-N-Phenyl-P-Phenylenediamine 0.1	Solid petroleum jelly
33	Ketoconazole 5	Solid petroleum jelly
34	Methyldibromoglutaronitrile 0.5	Solid petroleum jelly
35	Glutaraldehyde 0.5	Solid petroleum jelly
36	Hydroquinone 1	Solid petroleum jelly
37	Promethazine 1	Solid petroleum jelly
38	Sesquiterpene Lactone Mix	Solid petroleum jelly
	*Allantolactone* 0.033	
	*Costunolide* 0.033	
	*Dehydrocostus lactone* 0,033	
39	Decyl Glucoside 5	Solid petroleum jelly
40	Caine mix II	Solid petroleum jelly
	Lidocaine 5	
	Tetracaine 2.5	
	Dibucaine 2.5	
41	Bacitracin 5	Solid petroleum jelly
42	Linalool Hydroperoxide 1	Solid petroleum jelly
43	Bacitracin 20	Solid petroleum jelly
44	Oleamidopropyl Dimethylamine 0.1	Water
45	Chlorhexidine 0.5	Water
46	Methylisothiazolinone 0.2	Water
47	Formaldehyde 2	Water
48	Cocamidopropyl betaine 1	Water
49	Amerchol® L101 100	as is
50	Kathon® CG 0,5	Water

a Current substances are shown in light blue, and new substances in dark blue.

The PT was performed according to the standard established by the ICDRG, considering day 1 as the day of PT application, day 2 as the time of removal and first reading of the tests (at 48 h), and day 3 as the day of the final reading, after 96 hours of test application.^11^ A new evaluation, with a delayed reading (120 h), could be performed, according to clinical decision, and that would be day 4. The following standardized reading was established: negative test (-), doubtful test (?), weakly positive test (+), strongly positive test (++), very strongly positive test (+++).

Cases with the possibility of a positive test due to “excited skin syndrome (ESS)” were excluded, according to established criteria.^12^

Clinical relevance was defined as the affirmative relationship between the response obtained in the test reading and the patient's current contact with the substance (the material containing it) causing the dermatosis. A positive test may still have prior relevance, that is, refer to a past contact, but not related to the current dermatosis.^1^

At the end of each test, patients were informed of the results and kept under follow-up at the Services.

For the final configuration of the proposal for the new Brazilian standard series (adults), substances that reached a minimum of 1% positivity were considered,^11^ and certain substances that are currently shown to be important through other studies.^6^, ^10^

### Data analysis

The collected data were stored in a standard Microsoft Excel® spreadsheet. Each center individually stored its data, and the spreadsheets from all centers were compiled into a single database by the coordinating center.

Univariate description was performed using SPSS (Statistical Package for the Social Sciences) version 20.0. Quantitative variables were assessed for normality using the Shapiro-Wilk test and were described by the mean and standard deviation in the presence of normality. Categorical variables were described by frequencies and percentages, and positivity frequencies were shown with the 95% confidence interval for proportions expressed as a percentage, calculated using Fisher's exact test. To assess the association between different factors and substance positivity, a bivariate analysis was performed, using the Chi-square test for factors with three or more categories or Fisher's exact test for comparisons in pairwise tables and in the presence of expected values ​​less than 5. The significance level was set at 5%.

## Results

**Figure 1:**
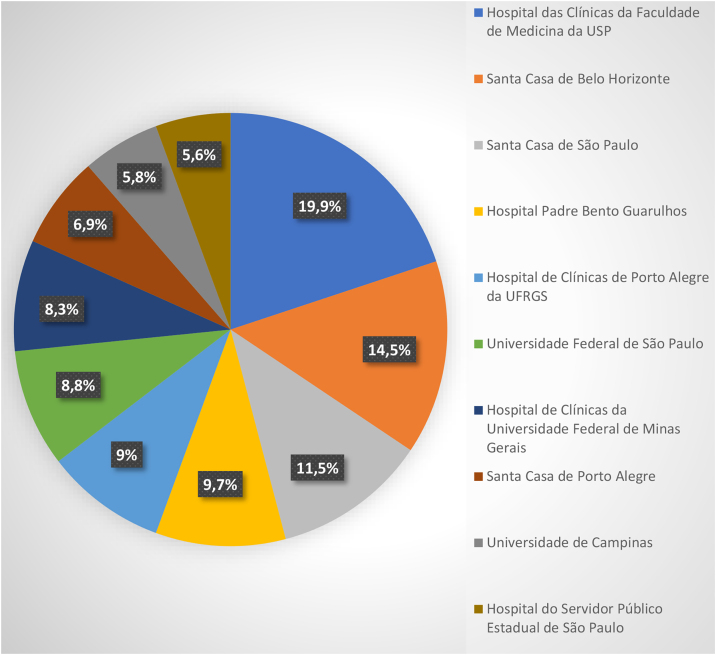
Participating centers: Dermatology Services accredited by the Brazilian Society of Dermatology (SBD) and the number of research participants. (2023‒2025).

The total number of participants included in the study was 567, distributed across the ten centers (Fig. 1).

The mean age was 53.3 years (standard deviation of 15.6 years), and females predominated, representing 74.3% of the participants. All phototypes were represented, with emphasis on types II (19.9%), III (34.3%), and IV (26.6%). A personal history of atopic disease (respiratory and/or cutaneous) was present in 243 participants (43.3%). Regarding occupation, 24% worked in the cleaning services area; 7.3% were health professionals; 7.2% worked in construction and 3.5% were professionals in the aesthetic care area; other occupations involved 57.1% of the remaining participants (Table 3).

**Table 3:** General characteristics of the sample from the Brazilian multicenter study (n = 567, Department of Allergy and Occupational Dermatoses of the Brazilian Society of Dermatology, 2023-2025).

Characteristics	Descriptive measurements
**Age** (in years; mean ± standard deviation)	aMean: 53.3 ± 15.6
**Sex**	
Female, n (%)	421 (74.3)
**Phototype,** n (%)	
I	21 (3.7)
II	112 (19.9)
III	193 (34.3)
IV	150 (26.6)
V	76 (13.5)
VI	11 (2.0)
Loss of information	4 (0.07)
**Personal history of atopy,** n (%)	
Yes	243 (43.3)
Loss of information	6 (0.17)
**Occupation,** n (%)	
Cleaning services / Homemaker	119 (21.0)
Office services / Teacher / Student	80 (14.1)
Retired	61 (10.8)
Healthcare sector	44 (7.8)
Mechanic	37 (6.5)
Construction services	30 (5.3)
Shopkeeper	26 (4.5)
Cook	25 (4.4)
Hairdresser / Manicurist	18 (3.2)
Tailor	13 (2.3)
Rural worker	13 (2.3)
Other occupations	4 (0.7)
No information	97 (17.1)
**Topography of Contact Dermatitis,** n (%)	
Hands	225 (39.7)
Upper limbs (except hands)	156 (27.5)
Chest	154 (27.2)
Face	148 (26)
Lower limbs (except feet)	120 (21.2)
Feet	114 (20.1)
Cervical region (neck)	87 (15.0)
Abdomen	63 (11.1)
Scalp	61 (10)
Gluteal region	23 (4.1)
**Overall positivity of patch tests,** n (%)	393 (69.3)
**Relevance of tests,** n (%)	272 (70.2)
Loss of information, n (%)	180 (31.7)

a Shapiro-Wilk test: used to assess normality.

In the topographic distribution of dermatitis, it was found that 39.7% affected the hands; 27.5% other regions of the upper limbs; 27.2% the chest; 26% the face; 21.2% the lower extremities other than the feet; 20.1% the feet; 15% the cervical region; 11.1% the abdomen; 10% the scalp and 4.1% the gluteal region (the sum exceeds 100% because several patients had the dermatosis in more than one location). Table 4 summarizes the main clinical and demographic characteristics of the studied sample.

**Table 4:** Prevalence of sensitizations to the allergens analyzed in the study (n = 567, Department of Allergy and Occupational Dermatoses of the Brazilian Society of Dermatology, 2023-2025).

Substance	n (%)	95%CI^a^
Nickel sulfate	164(28.9)	25.2‒32.9
Methylisothiazolinone	68 (12.0)	9.4‒15.0
Formaldehyde	59 (10.4)	8.0‒13.2
Cobalt chloride	58 (10.2)	7.9‒13.0
Fragrance MIX I	40 (7.1)	5.1‒9.5
Kathon CG®	37 (6.5)	4.6‒8.9
Paraphenylenediamine	36 (6.4)	4.5‒8.7
Neomycin	33 (5.8)	4.0‒8.1
Disperse blue 106	29 (5.1)	3.5‒7.3
Methyldibromoglutaronitrile	27 (4.8)	3.2‒6.9
Amerchol® L101	26 (4.6)	3.0‒6.7
Potassium dichromate	24 (4.2)	2.7‒6.2
Balsam of Peru	21 (3.7)	2.3‒5.6
Ketoconazole 1	18 (3.2)	1.9‒5.0
Caine mix I	16 (2.8)	1.6‒4.5
Carba mix	16 (2.8)	1.6‒4.5
Bacitracin 5%	16 (2.8)	1.6‒4.5
Tosylamide formaldehyde resin	15 (2.7)	1.5‒4.3
Decyl glucoside	15 (2.7)	1.5‒4.3
Cocamidopropyl betaine	15 (2.7)	1.5‒4.3
Thiuram MIX	14 (2.5)	1.4‒4.1
Ketoconazole 5%	14 (2.5)	1.4‒4.1
Bacitracin 20%	14 (2.5)	1.4‒4.1
Fragrance mix II%	13 (2.3)	1.2‒3.9
Hydroxyethyl methacrylate	12 (2.1)	1.1‒3.7
Colophony	10 (1.8)	0.9‒3.2
Bronopol	9 (1.6)	0.7‒3.0
Propylene glycol	6 (1.1)	0.4‒2.3
Glutaraldehyde	6 (1.1)	0.4‒2.3
Tertiary p-butylphenol	5 (0.9)	0.3‒2.1
PPD mix	5 (0.9)	0.3‒2.1
Ethylenediamine	5 (0.9)	0.3‒2.1
Mercapto MIX	5 (0.9)	0.3‒2.1
Promethazine	5 (0.9)	0.3‒2.1
Paraben MIX	4 (0.7)	0.2‒1.8
*Compositae* MIX 2,5%	4 (0.7)	0.2‒1.8
N-isopropyl-n-phenyl	4 (0.7)	0.2‒1.8
Sesquiterpene lactone mix	4 (0.7)	0.2‒1.8
Caine mix II	4 (0.7)	0.2‒1.8
Irgasan	3 (0.5)	0.1‒1.5
Imidazolidinyl urea	3 (0.5)	0.1‒1.5
DMDM hydantoin	3 (0.5)	0.1‒1.5
*Compositae* MIX 5	3 (0.5)	0.1‒1.5
Linalool hydroperoxide	3 (0.5)	0.1‒1.5
Oleamidopropyl dimethylamine	3 (0.5)	0.1‒1.5
Hydroquinone	2 (0.4)	0.1‒1.3
Limonene hydroperoxide	1 (0.2)	0‒1.0
Chlorhexidine	1 (0.2)	0‒1.0
Bisphenol A epoxy resin	‒	0‒0.7
Phenoxyethanol	‒	0‒0.7

a CI, 95% Confidence Interval for prevalence using Fisher's exact method.

Among the total of 567 patients tested, 393 (69.3%) were positive for at least one of the 50 tested substances and 132 (23.3%) patients were positive for three or more substances, not constituting excited skin syndrome, given the clinical evidence of exposure (polysensitization).

Of the 387, 272 positive tests (current or past) were considered relevant (this information was missing for 180 patients), meaning the relevance was 70.3%.

When evaluating relationships between variables, a significant association (p < 0.05) was found between male sex and the substances potassium dichromate, ketoconazole, colophony, and cobalt chloride; between female sex and nickel sulfate; between the age group up to 30 years and the substances propylene glycol and chlorhexidine; and between 30-60 years and thiuram mix.

The allergens that showed a sensitization frequency equal to or greater than 1% were: nickel sulfate (28.9%), methylisothiazolinone (12%), formaldehyde (10.4%), cobalt chloride (10.2%), fragrance mix I (7.1%), Kathon CG® (6.5%), paraphenylenediamine (6.4%), neomycin (5.8%), disperse blue 106 (5.1%), Methyldibromo glutaronitrile (4.8%), Amerchol® L101 (4.6%), potassium dichromate (4.2%), balsam of Peru (3.7%), ketoconazole 1% (3.2%), Caine mix I (2.8%), carba mix (2.8%), bacitracin 5% (2.8%), Tosylamide (2.7%), decyl glucoside (2.7%), cocamidopropyl betaine (2.7%), thiuram mix (2.5%). Ketoconazole 5% (2.5%), bacitracin 20% (2.5%), fragrance mix II (2.3%), hydroxyethyl methacrylate (2.1%), colophony (1.8%), Bronopol (1.6%), propylene glycol (1.1%), and glutaraldehyde (1.1%) (Table 4).

Of the 30 substances that currently make up the standard series of patch tests (Table 1), eight did not reach 1% positivity in this study: with 0.9% were tertiary p-butylphenol, PPD (mix), ethylenediamine, Mercapto (mix), promethazine; with 0.7% were parabens (mix); with 0.5%, Irgasan; with 0.4% hydroquinone; and with 0% positivity, epoxy resin.

Based on these results and the discussion presented below (which involves the literature and considers emerging allergens), the new Brazilian standard series for patch tests, as proposed, is as follows (Table 5).

**Table 5:** Brazilian Standard Series for Patch Tests – 2025: substances, concentrations and vehicles.

Brazilian Standard Series for Patch Tests GBEDC – 2025			
N.	Substance	Concentration	Vehicle
1	Nickel sulfate	5%	Solid petroleum jelly
2	Linalool hydroperoxide	1%	Solid petroleum jelly
1	Imidazolidinylurea	2%	Solid petroleum jelly
4	Cobalt Chloride	1%	Solid petroleum jelly
5	Fragrance mix I	14%	Solid petroleum jelly
	Cinnamic alcohol 2%		
	Alpha-amyl cinnamaldehyde 2%		
	Eugenol 2%		
	Isoeugenol 2%		
	Geraniol 2%		
	Hydroxycitronellal 2%		
	Oak moss absolute 2%		
6	DMDM hydantoin	1%	Solid petroleum jelly
7	Bisphenol A epoxy resin	1%	Solid petroleum jelly
8	Amerchol® L101	100%	As is
9	Ketoconazole	1%	Solid petroleum jelly
10	Caine MIX	10%	Solid petroleum jelly
	Benzocaine 5%		
	Tetracaine 2,5%		
	Dibucaine 2,5%		
11	Disperse blue 106	1%	Solid petroleum jelly
12	Methyldibromoglutaronitrile	0.5%	Solid petroleum jelly
13	Potassium Dichromate	0.5%	Solid petroleum jelly
14	Balsam of Peru	25%	Solid petroleum jelly
15	Paraben Mix	15%	Solid petroleum jelly
	Methylparaben 3%		
	Ethylparaben 3%		
	Propilparabeno 3%		
	Butylparaben 3%		
	Benzylparaben 3%		
16	Thiuram mix	1%	Solid petroleum jelly
	Tetramethylthiuram disulfide 0.5%		
	Tetramethylthiuram monosulfide 0.5%		
17	Tosylamide	10%	Solid petroleum jelly
18	Decyl glucoside	5%	Solid petroleum jelly
19	Carba MIX	3%	Solid petroleum jelly
	Diphenylguanidine 1%		
	Zinc dimethyldithiocarbamate 1%		
	Zinc diethyldithiocarbamate 1%		
20	Bacitracin	5%	Solid petroleum jelly
21	Fragrance Mix II	14%	Solid petroleum jelly
	Coumarin 2.5%		
	Farmesol 2.5%		
	Lyral 2.5%		
	Citral 1%		
	Citronellal 0,5%		
	Aldehydohexyl cinnamyl 5%		
22	Hydroxyethyl methacrylate	2%	Solid petroleum jelly
23	Colophony	20%	Solid petroleum jelly
24	Propylene glycol	10%	Solid petroleum jelly
25	Bronopol	0.5%	Solid petroleum jelly
26	Tertiary p-butylphenol	1%	Solid petroleum jelly
27	Glutaraldehyde	0.5%	Solid petroleum jelly
28	PPD MIX	0.4%	Solid petroleum jelly
	N-isopropyl, n-phenyl, paraphenylenediamine 0.2%		
	N-N diphenyl, paraphenylenediamine 0.2%		
29	Ethylenediamine	1%	Solid petroleum jelly
30	Mercapto MIX	2%	Solid petroleum jelly
	Mercaptobenzothiazole 0.5%		
	Dibenzothiazole disulfide 0.5%		
	Morpholinylmercaptobenzothiazole 0.5%		
	N-cyclohexyl 2 benzothiazole sulfonamide 0.5%		
31	Promethazine	1%	Solid petroleum jelly
32	*Compositae* MIX	2.5%	Solid petroleum jelly
	*Anthemis nobilis* extract 0.6%		
	*Chamomilla recutita* extract 0.6%		
	*Achillea millefolium* extract 0.5%		
	*Tanacetum vulgare* extract 0.5%		
	*Arnica montana* extract 0.25%		
	*Parthenolide* 0.05%		
	*j-allantolactone* 0.03%		
	*Costunolide* 0.03%		
	*Dehydrocostus lactone* 0.033%		
33	Neomycin	20%	Solid petroleum jelly
34	Sesquiterpene lactone MIX	0.1%	Solid petroleum jelly
	Allantolactone 0.033%		
	Costunolide 0.033%		
	Dehydrocostus lactone 0,033%		
35	Paraphenylenediamine	1%	Solid petroleum jelly
36	Limonene hydroperoxide	0.3%	Solid petroleum jelly
37	Methylisothiazolinone	0.2%	Water
38	Cocamidopropyl betaine	1%	Water
39	Chlorhexidine	0.5%	Water
40	Formaldehyde	2%	Water

Of the 50 substances tested, those not included in the new standard series are: phenoxyethanol, hydroquinone, Irgasan, oleamidopropyl dimethylamine, N-Isopropyl-n-phenyl, Caine Mix II, bacitracin 20%, ketoconazole 5%, Compositae 5%, and Kathon CG®.

## Discussion

The prevalence and incidence of positivity to different allergens vary over time, following changes in population habits, industrial transformations, and the availability of sensitizing agents.^3^, ^4^, ^10^, ^13^ In 2000, the GBEDC proposed the current Brazilian standard series of epicutaneous tests.^9^ Since then, important changes in the sensitization profile have been observed, not only in Brazil but throughout the world.

It is important to emphasize that in the present study, cases of excited skin syndrome were excluded, and those considered to have polysensitization were maintained. ESS, also called “angry back syndrome,” refers to a state of transient cutaneous hyper-reactivity that occurs in patients with active or very reactive eczema, submitted to epicutaneous tests. In this situation, the skin is in an exacerbated inflammatory state, with amplified immunoinflammatory responses, leading to the appearance of false-positive reactions.

A polysensitized patient is one who truly has sensitivity due to an immunological reaction to multiple agents, with several positive patch tests, generally with allergens unrelated to each other. This condition reflects a broad cutaneous immunological reactivity, resulting from cumulative exposure to various sensitizing agents and, in some cases, from cross-reactive immunological mechanisms between structurally similar compounds.

The diagnosis of polysensitization should be differentiated from ESS, in which multiple positive reactions may occur transiently, without representing a true specific allergy. In contrast, in the polysensitized patient, positive responses tend to be reproducible, clinically relevant, and persistent in repeated tests.^12^

In the study published in 2000,^9^ most patients were female (62.5%), a finding that was repeated in the present study, with an even higher proportion (74.3%). At that time, 62.0% of the individuals showed sensitivity to at least one substance, while in the current study, this percentage was 69.3%. Regarding age range, in 2000, patients were predominantly between 20 and 49 years old, whereas in the present case series, the average age was higher, at 53.3 years.

Among the 567 patients tested in this study, information about the profession was not obtained for 97; 61 were retired, and four did not have a well-defined professional activity. Among the 405 patients with the professions mentioned, cleaning service workers/homemakers were the most common (21%). Despite the wide range of occupations found, it is noteworthy that many participants performed wet professional activities, one of the relevant factors in the predisposition to the development of contact dermatitis.

Regarding relevance, a clinical concept that establishes the relationship between proven sensitization and known current or past exposure, there was a loss of 31.7% of the information, limiting the analysis of this aspect. However, of the 387 patients with reported data, 70.3% (272) had relevant tests, which demonstrates the importance of applying tests for the individual etiological search for allergic contact dermatitis. Furthermore, all the data in the study refer to the 567 patients and not to the relevant ones, since the general sensitization to allergens is considered for the assembly of the series.

Of the 30 substances in the standard series defined in 2000,^9^ 19 remained: nickel sulfate, formaldehyde, carba mix, fragrance mix I, paraphenylenediamine, neomycin, cobalt chloride, potassium dichromate, balsam of Peru, thiuram mix, Mercapto mix, colophony, propylene glycol, PPD mix, ethylenediamine, promethazine, epoxy resin, tertiary p-butylphenol and parabens; and 11 were excluded: anthraquinone, hydroquinone, Irgasan, lanolin, Kathon CG®, benzocaine, Quaternium, quinoline, nitrofurazone, thimerosal and turpentine. In the development of the new series, 21 substances were included: sesquiterpene lactone, Amerchol® L101, ketoconazole, Caine mix, methyl bromo glutaronitrile, cocamidopropyl betaine, Tosylamide, decyl glucoside, disperse blue 106, bacitracin, fragrance mix II, hydroxyethyl methacrylate, Bronopol, glutaraldehyde, methylisothiazolinone, Compositae mix, DMDM ​​hydantoin, limonene, linalool, imidazolidinyl urea, and chlorhexidine.

Although the reference for inclusion in the new series was defined as 1% positivity, the authors included substances with lower rates. This decision aimed to ensure a series with a larger number of substances (of the 40 tested substances, 29 reached 1%), not excluding allergens currently considered emerging and of great clinical importance, considering data from the Brazilian systematic review, which analyzed more than two decades of the performance of the current standard series tests^10^ – and international trends.

Thus, the following substances were retained for the new series, with less than 1% positivity, with their synthetic justification: ethylenediamine (amine widely used in various industrial applications), tertiary p-butylphenol (important in occupational medicine), Mercapto mix (important in occupational medicine), promethazine (antihistamine extensively used in Brazil and sold over the counter), Compositae (marker of plant sensitivity), neomycin (main topical antibiotic available in public pharmacies in Brazil), parabens (preservative in frequently used personal products, as well as food and medicines), cobalt chloride (ubiquitous metal, and important in occupational medicine), and epoxy resin (frequently used as a component of coatings). The following substances were also included with a frequency of less than 1%, due to their emergence in the market, and have been part of the European series since 2019^14^, ^15^: linalool hydroxyperoxide and Limonene hydroxyperoxide (both are terpenes, natural organic compounds derived from plants, herbs, and citrus fruits, used for their aroma-enhancing properties in fragrances and other products). Imidazolidinyl urea and chlorhexidine are substances that showed positivity rates of less than 1%, but are very important in the cosmetics industry, and were included in the new standard series (both are part of the cosmetics test series, migrating from the status of a complementary option with this repositioning).^14^, ^15^

If the systematic review by Bueno ALA, Aguzzoli NHG, Bonamigo RR,^10^ is used, which analyzed 22 years of Brazilian publications and 4703 patients, the substances that were included with less than 1% in the present study appear with positivity rates between 2000-2022, as follows: cobalt chloride – 10.7%, neomycin – 7.1%, ethylenediamine – 4%, Promethazine – 2.5%, paraben – 2.4%, epoxy resin – 1.8%, Mercapto-mix – 1.8%, tertiary p-butylphenol – 0.9%. These substances have historical and current importance. The authors intend to conduct a new evaluation of the proposed series in five years to analyze these substances that were included without meeting the 1% quota.

Alternatively, Kathon CG® was removed from the new series, despite its positivity being above 1%, because it integrates the allergen methylisothiazolinone, which had a 12% positivity rate in this series.

The substance groups will be detailed individually below.

### Allergens

#### Metals

Regarding the allergens, in 2000 nickel sulfate was the most prevalent one (25.1%), a position that remained in the present study, with a rate of 28.9%.^9^ A recent systematic review of Brazilian studies showed a prevalence of 31.8% between 2000 and 2022.^10^ Data from the American Contact Dermatitis Society (ACDS) corroborate these findings, with similar positivity to nickel (24.9%).^14^ In contrast, a recent study conducted in Israel revealed a higher prevalence (41%).^15^ Additionally, a European multicenter study conducted in 13 countries (n = 7,675; 2019–2020), coordinated by the European Surveillance System for Contact Allergies (ESSCA) and the European Society of Contact Dermatitis (ESCD), recorded an overall prevalence of nickel sensitization of 19.8%, with wide variation between countries (30.3% in Austria vs. 13.1% in the United Kingdom).^16^ This heterogeneity is partly attributed to the adoption of the Nickel Directive, which came into force in 2000, was revised in 2004 and incorporated into European regulations in 2006, resulting in a significant decline in sensitization in several countries, such as Germany, the United Kingdom, Italy, Sweden and Denmark.^15^

In the same European study, the prevalence of sensitization to other metals was 6.2% for cobalt and 4.4% for chromium.^16^ In Brazil, in 2000, the values ​​found were 11% for cobalt and 8.1% for chromium.^9^ In the present study, values of 10.2% and 4.2% were found for cobalt and chromium, respectively. In the 2025 Brazilian systematic review, the values ​​were 10.7% and 9%, respectively.^10^ In the ACDS study, the rates were 9.2% for cobalt and 3% for dichromate.^14^

#### Preservatives

Among the preservatives, Methylisothiazolinone (MI) stood out as one of the main sensitizers in the present study. In 2013, it was named “allergen of the year” by the ACDS due to the outbreak of contact dermatitis it triggered.^17^, ^18^ In the most recent North American studies, positivity reached 15.3% between 2017 and 2018, decreasing to 11.5% between 2021 and 2022, suggesting stabilization.^14^ In Europe, the peak occurred between 2013 and 2014, with 8.7% positivity; however, regulatory measures – such as the removal of MI from leave-on products and the limitation to 15 ppm in rinse-off products – reduced positivity to 2.9% in 2024.^19^ In Brazil, ANVISA Resolution RDC N. 528/2021 prohibited the use of MI and Kathon CG® (MI and methylchloroisothiazolinone) in leave-on formulations.^20^ Despite this, in the present study, MI was still the second most prevalent sensitizer, with a positivity rate of 12%.

In the standard Brazilian series created in 2000 and still used, MI is tested in association with methylchloroisothiazolinone as a component of Kathon CG®. In 2000, the positivity rate for Kathon was 2.2%, whereas in the 2025 systematic review, it was 3.2%.^10^ In the present study, the positivity rate for Kathon CG® was 6.5%, reinforcing the need to test MI alone, a strategy that increases the detection of positive cases.^21^, ^22^

Formaldehyde (formalin) was the third most prevalent allergen, with a rate of 10.4%, higher than the 5.5% reported in the North American group.^14^ In addition to acting as a preservative, formaldehyde is widely used in disinfectants, glues, construction materials, and hair straightening products, such as “progressive straightening,”^23^, ^24^ often in association with glutaraldehyde, of which positivity prevalence was 1.1% in this study.

Three formaldehyde releasers – toluenesulfonamide formaldehyde resin (Tosylamide), Bronopol, and tertiary p-butylphenol – showed positivity of 2.7%, 1.6%, and 0.9%, respectively.

Tosylamide shows a consistent downward trend: an Australian study reported positivity of 1.9% in 2005, while in 2017, none of the 364 patients evaluated showed sensitization.^25^ In a Brazilian center, the prevalence decreased from 16% to 2.1% in the last decade.^26^ In the present study, its positivity was 2.7%. Despite this reduction, national rates are still relatively high, possibly due to the intense use of nail polish in Brazil, which ranks second in the world in terms of consumption volume.^26^

Bronopol, another antimicrobial preservative, showed a significant increase in recent North American studies,^14^ while tertiary p-butylphenol showed a progressive decline: an Italian study (1997–2021) revealed a decrease from 1.9% in 2000 to 0.55% in 2021, attributed to the gradual replacement of resins in glues by acrylates and epoxy.^27^

Another cosmetic preservative, Methyldibromo glutaronitrile, showed a positivity of 4.8% in the present study. This agent is frequently used in combination with phenoxyethanol. In the United States, sensitization to this combination decreased from 6% between 1998 and 2000 to 2.5% in 2017–2018. The same study highlighted, however, the high frequency of weak reactions (63.3%) and the low clinical relevance (3% definite and 19.3% probable), emphasizing the need for cautious interpretation of results.^28^

Colophony, also called rosin, used as a natural preservative in soaps and depilatory waxes, showed a positivity of 1.8% in the present study, a value lower than the 2.9% reported in the 2025 Brazilian systematic review that evaluated the allergens of the series proposed in 2000.^10^ In the New Zealand standard series study, positivity was higher, at 5.1%^29^; while in the United States, it was 2.5%^14^; data that reinforce the relevance of this allergen and support its maintenance in the standard series.

Propylene glycol showed identical positivity in this study and in the 2025 Brazilian systematic review, both at 1.1%.^10^ It had been elected “allergen of the year” in 2018.^30^ Despite its low prevalence, it is considered clinically significant, as it tends to produce weak reactions that should still be considered.^31^

Parabens were considered “non-allergens” in 2019^32^ due to their low allergenicity. In this study, positivity was 0.7%, similar to the North American rate of 0.5%.^14^ In Portugal, a 0.49% rate was observed, with a clinical relevance of 0.32%.^33^ The 2025 Brazilian review reported 2.4% sensitization, although without an assessment of clinical relevance.^18^ Given this fact, it was decided to keep parabens in the standard series.

#### Emollients and surfactants

Amerchol® L101 is a mixture of lanolin and mineral oil. Studies show that mineral oil enhances the absorption of lanolin, increasing the accuracy of patch tests.^34^ In this study, Amerchol® L101 was used as a substitute for pure lanolin. Lanolin, derived from the sebaceous gland of sheep, is widely used in cosmetics and topical medications, with sensitization rates ranging from 1.2% to 6.9%. In the present study, the frequency of positive tests for Amerchol® was 4.6%, higher than that observed in some previous national studies, in which lanolin showed rates between 0.4% and 2.8%.^35^, ^36^, ^37^ Data from the American group show a frequency of 2.3%.^14^ This result reinforces that Amerchol L101 may be a more sensitive marker to identify patients sensitized to lanolin in our setting.

Decyl glucoside is a non-ionic surfactant widely used in cosmetics (shampoos, sunscreens, fragrances, deodorants) and cleaning products. Cases of Allergic Contact Dermatitis (ACD) related to this substance have been described since 2003, with prevalence rates ranging from 1% to 5%.^38^, ^39^ In the present study, the sensitization frequency was 2.7%. Decyl glucoside was not previously tested in the Brazilian standard series, which highlights the relevance of its inclusion.

Cocamidopropyl betaine is an amphoteric surfactant, introduced in the 1970s as a substitute for sodium lauryl sulfate and other anionic surfactants, due to its lower irritant potential, especially ocular. It is found in shampoos, conditioners, shower gels, liquid detergents, toothpastes, mouthwashes, and other cosmetics. Sensitization rates reported in the literature range from 3% to 7.2%.^40^ In the present study, the frequency was 2.5%, a lower value, but one that confirms its role as a relevant allergen in personal care products.

#### Fragrances

Allergic contact dermatitis to fragrances remains one of the main causes of sensitization worldwide, representing the second or third most frequent cause, after metals and together with preservatives.^41^ The present study found a prevalence of positive reactions of 7.1% for fragrance MIX I (FM I), 3.7% for balsam of Peru (*Myroxylon pereirae*), 2.3% for fragrance MIX II (FM II), 1.8% for colophony, 0.5% for linalool hydroperoxide and 0.2% for limonene hydroperoxide. These results are consistent with international data and reinforce the need for continuous updating of standard patch test series.

European studies show similar prevalence rates: a recent systematic review showed an average sensitization of 6.8% for FM I and 3.6% for FM II, values ​​close to those observed in this study.^42^ Balsam of Peru showed 3.7%, slightly lower than the rates of the North American Contact Dermatitis Group (NACDG; 7%) and European groups (6.62%).^14^, ^16^ Colophony, included in some series as an additional marker of fragrance allergy, was discussed above and showed a positivity of 1.8%.

In contrast, linalool and limonene hydroperoxides showed very low prevalence (0.5% and 0.2%, respectively) in this study. This discrepancy may reflect the instability of these substances, differences in the formulation of the allergens in the tests, and the variability of exposure in our country. In recent European and American series, the prevalence rates of sensitization to these compounds have been higher, especially in young populations.^14^, ^43^

FM I remains the most sensitive marker for detecting fragrance allergies. However, several studies demonstrate that its isolated use can underdiagnose patients. The inclusion of FM II and oxidative pre-haptens, such as linalool and limonene, has been proposed to increase diagnostic yield. The findings of the present study corroborate this need, at least in relation to FM II, since the current Brazilian standard series includes only FM I, Balsam of Peru, and colophony.^9^ Despite the low positivity identified in the present sample, the presence of linalool and limonene in cosmetics and hygiene products suggests that their inclusion in complementary series may be strategic for diagnosis in specific subgroups. Given all these situations, the authors chose to add them to the new standard series.

#### Topical Medications

Topical medications are frequent sources of CD, especially in individuals with dermatoses or chronic ulcers. Some of them are available without a prescription in our setting, such as neomycin and ketoconazole. Furthermore, there are formulations that combine different drugs, which increases the likelihood of simultaneous contact with multiple allergens.

Neomycin, an antibiotic from the aminoglycoside group, is a globally recognized allergen, which is why it is included in several standard series of patch tests. Scherrer et al. identified its presence in several products available in Brazil, including dermatological creams and ointments, ophthalmic, otologic, oral and nasal suspensions, antiseptic powders, and even vaccines.^44^ In the present study, neomycin was the drug with the highest frequency of positivity (33 cases; 5.8%), a finding similar to that of a 2025 North American study, where it is also marketed without restriction.^14^ In the Brazilian systematic review, an average increase of 1.41% in the frequency of positive tests for neomycin was demonstrated between 2000 and 2022.^10^ Another systematic review and meta-analysis on neomycin-related CD, also from 2025, analyzing 70 international studies, showed a prevalence of 3.2% in adults and 4.3% in children. The highest frequencies were observed in the United States (6.4%), and the lowest in the European Union (2.5%) and the Middle East (0.8%), regions with more restrictive regulations on its use.^45^ These data reinforce the importance of maintaining neomycin as a component of the standard series.

Bacitracin, a topical antibiotic, is available in our setting only in combination with neomycin. It can cause allergic contact dermatitis, contact urticaria, and even anaphylaxis.^46^ It was positive in 2.5% (14 cases), a frequency higher than that observed in European studies, but lower than that recorded in the United States (6.4–8.3%), where bacitracin is sold freely, as well as in Brazil.^47^ There are no national comparative data, as the substance was not included in the allergen series previously used in the country.

Ketoconazole, an imidazole antifungal indicated for the treatment of superficial mycoses, is widely used in our environment. It is present in combination with corticosteroids in creams and ointments (sold without prescription), in shampoos, and is also one of the few antifungals available in the public health system. This greater exposure makes it a more relevant allergen in Brazil compared to other countries. In the present study, the frequency of positive tests was 3.2% (18 cases), a value higher than that observed in a previous Brazilian study, carried out in a single hospital center between 2010 and 2017, which found a positivity rate of 2.3%.^48^ These data support its inclusion in the standard series for evaluation in different regions of the country.

Topical anesthetics are widely used in various health fields, available in presentations such as creams, ointments, eye drops, ear drops, lozenges, and sprays. They are used in dermatology, ophthalmology, otorhinolaryngology, proctology, gynecology, urology, and dentistry, and are also found in over-the-counter products for minor burns. Absorption is favored when applied to mucous membranes or previously damaged skin. Benzocaine has always been considered a marker of CD by topical anesthetics; however, its effectiveness began to be questioned in the 1980s.^16^ In 2019, the European series incorporated the mixture of Caines, as it showed a higher positivity rate than benzocaine alone, especially due to the presence of dibucaine.^16^, ^47^ The American series, however, maintains separate testing of lidocaine and benzocaine.^14^ The expert group in this study chose the Caine Mix, which includes benzocaine, tetracaine, and dibucaine (cinchocaine). A frequency of 2.8% (16 cases) was obtained, higher than the average observed in European studies (1.56%),^16^ possibly related to differences in prescribing habits.

Promethazine, a phenothiazine derivative with antihistamine properties, is used in our setting mainly in topical form, but also intramuscularly in emergency services. It can also be part of oral formulations combined with analgesics (e.g., Lisador®). The substance can cause CD, photoallergic CD, and photosensitivity reactions after systemic use (pharmacodermia).^49^, ^50^ Although the frequency of positive tests was low in the present study (0.7%), the expert group recommended its maintenance in the series, as it is a product frequently used in our setting and potentially associated with serious reactions, the diagnosis of which should not be neglected.

#### Rubber Allergens

Rubber allergens play an important role in the investigation of ACD, especially in occupational contexts. Manifestations mainly affect hands (use of gloves) and feet (footwear).^51^ In the present study, hands and feet were affected in 39.7% and 20.1% of patients, respectively. It was observed that 21% worked in cleaning/household services; 6.9% were health professionals and 5.1% worked in construction; 32.5% belonged to occupations with high exposure to rubber.

Carba mix and thiuram mix are structurally related, can convert to each other by oxidation/reduction reactions and are often co-positive. Thiurams may be better markers of sensitization to dithiocarbamates than the carbamates themselves.^16^, ^52^

The carba mix showed a positivity rate of 2.8%, lower than the Brazilian systematic review (4.7%), but slightly higher than the North American data (2.5%) and data from European countries that use the True Test (2.4%), but it is not tested in the European series.^10^, ^14^, ^16^

The thiuram mix showed 2.5% positivity, similar to tests with the European standard series (2.34%) and in the North American group (NACDG – 3%), but with a lower frequency in the countries tested with the True Test (1.63%).^10^, ^14^, ^16^

Thus, data from the current study were very similar to the European and North American data, with a decrease in the frequency of the carba mix, despite carbamates being irritants and causing false positive reactions.^10^, ^14^, ^16^, ^51^

The antioxidants used in the manufacture of black rubber constitute the PPD mix, included in the standard series. In the Brazilian systematic review, its frequency was 4.3%, substantially higher than that observed in the present study (0.9%). This last result, however, was similar to the North American data (0.4%) and those obtained in Europe (0.79% in the standard series and 0.88% in the True Test).^10^, ^14^, ^16^

Paraphenylenediamine (PPDA) is the main allergen present in permanent hair dyes, acting as an oxidizing agent. It can also be present in henna tattoos, eyelash makeup, elastic bands, and rubber products.^53^ In the present study, the sensitization frequency was 6.4%, similar to that reported in the Brazilian systematic review (7.8%), but both above the European (between 3.6%–3.87%) and North American (5%) reviews. In a national study with 120 patients sensitized to rubber, 49 (40.8%) tested positive for PPDA, with 27 cases related to hair dye. These findings confirm the relevance of PPDA as one of the main cosmetic allergens and support its maintenance in standard series.^10^, ^14^, ^16^, ^51^

Mercapto mix showed positivity of 0.9% in this study, approaching the European results (0.72% in the standard series and 0.43% in the True Test), but lower than that reported by the Brazilian systematic review (1.8%).^10^ The North American group, in turn, does not test for Mercapto mix, including only mercaptobenzothiazole, considered the true hapten represented in the mixture.^16^, ^51^

Ethylenediamine was detected in 0.9% of patients, a value lower than that described in the Brazilian systematic review (4%).^10^ In contrast, its frequency was similar to that reported by the North American group (1.2%) and by European studies with True Test (0.88%), and will remain in the Brazilian series.

Hydroquinone showed a prevalence of 0.4% in this study, contrasting with the 2% in the Brazilian review. There are no mentions of this hapten in the European and North American studies.^10^, ^14^, ^16^ Therefore, it will not be included in the new Brazilian series.

#### Miscellaneous

Disperse blue 106 is a dye used in synthetic fibers such as nylon and polyester, mainly present in hosiery fabrics. Among textile dyes, it is recognized as a frequent sensitizer. In the present study, the sensitization frequency was 5.1%. In the international literature, the rates vary between 3% and 6%.^14^, ^54^, ^55^ This is an allergen that was not part of the standard Brazilian series, reinforcing the importance of its inclusion for a broader evaluation in the country.

Hydroxyethyl methacrylate (HEMA) was incorporated into the European series of contact tests in 2019, being the main agent implicated in cases of ACD due to artificial nails and long-lasting nail polish. Kocabas et al. (2024) reported sensitization rates of 3.9% in women and 1.0% in men.^56^ In the present study, the observed frequency was 2.1%. Recent studies by the North American group also confirm its relevance.^14^ These data reinforce the importance of including HEMA in the standard series to monitor the increasing exposure associated with aesthetic procedures.

Epoxy resin is an important, highly resistant industrial product widely used in various contexts, composing products for sealing, paints for markings. It can also be important in the electrical and technological industry, in circuit boards, encapsulation, fiberglass reinforcement, concrete, metals, wood, and flooring coating. It was retained in the new series due to its importance in occupational health, being positive above 1% in the Brazilian systematic review published by Bueno et al.^10^

This study has some limitations. Although it is a multicenter study, the sample may not represent all regions of the country equitably, as it was only conducted in the south and southeast of the country. Furthermore, the absence of an expanded series of fragrances may have underestimated the true prevalence of sensitization to emerging allergens. The analysis of clinical data may also be subject to inconsistencies in the documentation of occupational history or allergen exposure due to being multicenter. Finally, the lack of prospective follow-up limits the assessment of the clinical relevance of some positive reactions, especially for allergens with a low frequency of positivity.

Despite the limitations, this work has relevant strengths. The relatively large sample size (567 patients) and the participation of multiple centers allow a representative assessment of the sensitization profile in Brazil. Comparison with national data (particularly with the foundational study of the current series and with the 2025 systematic review) and international data makes it possible to contextualize the findings, identifying trends and regional differences.^9^, ^10^ The inclusion of emerging allergens, such as cocamidopropyl betaine, MI, some fragrance allergens, HEMA, and textile dyes, reinforces the usefulness of the study for updating the standard series of epicutaneous tests. Moreover, the inclusion of plant markers such as *Compositae* mix and sesquiterpene lactone contemplates the possibility of analyzing this topic in a country of continental proportions, with multiple vegetation types and a large proportion of rural population.

From its use in daily practice, new data will emerge, and more frequent reassessments of the new standard series will be necessary to prevent it from becoming outdated.

## Conclusions

The study indicated the need to reformulate the current series of 30 substances to be tested in the Brazilian standard series of patch tests, as 11 substances were evaluated as outdated.

The article proposes maintaining 19 substances from the current series and including 21 new substances, based on the statistical criteria of this study and the analysis of the literature, resulting in a new Brazilian standard series with 40 substances.

## ORCID ID

Renan Rangel Bonamigo: 0000-0003-4792-8466

Vitor Manoel Silva dos Reis: 0000-0001-5705-4104

Dulcilea Ferraz Rodrigues: 0009-0007-3599-0613

Rosana Lazzarini: 0000-0002-4893-3593

Mario Cezar Pires: 0000-0001-7587-8932

Renato Shintani Hikawa: 0000-0003-3319-5994

Ana Elisa Kiszewski: 0000-0002-6287-6302

Andrea Fernandes Eloy da Costa França: 0000-0003-1657-4570

Renata Heck: 0000-0003-2352-3915

Maria Antonieta Rios Scherrer: 0000-0001-7323-4260

Luciana Paula Samorano: 0000-0001-7077-8553

Isabella Campos Machado Cordeiro: 0009-0003-2663-9208

Ida Alzira Duarte: 0000-0001-6554-7005

Mariana de Figueiredo Silva Hafner: 0000-0001-8322-3856

Renata Marli Gonçalves Pires: 0000-0002-6699-0591

Lilith Sodré Eller: 0000-0001-6923-134X

Juliana Barros Rodrigues: 0009-0003-9582-1492

Daniela Benzano Bumaguin: 0000-0001-6293-8684

Ruppert Ludwig Hahnstadt: 0000-0002-7440-8549

## Research data availability

The entire dataset supporting the results of this study was published in this article.

## Financial support

This study received financial support from FDA Allergenics (Rio de Janeiro, RJ), through the provision of materials for the Patch Tests.

## Authors' contributions

Renan Rangel Bonamigo: Design and planning of the study; collection of data, or analysis and interpretation of data; statistical analysis; drafting and editing of the manuscript or critical review of important intellectual content; collection, analysis, and interpretation of data; effective participation in research orientation; intellectual participation in the propaedeutic and/or therapeutic conduct of the studied cases; critical review of the literature; approval of the final version of the manuscript.

Vanessa Barreto Rocha: Collection and acquisition of data; drafting and editing of the manuscript or critical review of important intellectual content; intellectual participation in the propaedeutic and/or therapeutic conduct of the studied cases; approval of the final version of the manuscript.

Vitor Manoel Silva dos Reis: Collection and acquisition of data; drafting and editing of the manuscript or critical review of important intellectual content; intellectual participation in the propaedeutic and/or therapeutic conduct of studied cases; approval of the final version of the manuscript.

Dulcilea Ferraz Rodrigues: Collection and acquisition of data; drafting and editing of the manuscript or critical review of important intellectual content; intellectual participation in the propaedeutic and/or therapeutic conduct of studied cases; approval of the final version of the manuscript.

Rosana Lazzarini: Collection and acquisition of data; drafting and editing of the manuscript or critical review of important intellectual content; intellectual participation in the propaedeutic and/or therapeutic conduct of the studied cases; approval of the final version of the manuscript.

Mario Cezar Pires: Collection and acquisition of data; drafting and editing of the manuscript or critical review of important intellectual content; intellectual participation in the propaedeutic and/or therapeutic conduct of the studied cases; approval of the final version of the manuscript.

Renato Shintani Hikawa: Collection and acquisition of data; drafting and editing of the manuscript or critical review of important intellectual content; intellectual participation in the propaedeutic and/or therapeutic conduct of the studied cases; approval of the final version of the manuscript.

Ana Elisa Kiszewski: Collection and acquisition of data; drafting and editing of the manuscript or critical review of important intellectual content; intellectual participation in the propaedeutic and/or therapeutic conduct of the studied cases; approval of the final version of the manuscript.

Andreia Fernandes Eloy da Costa França: Collection and acquisition of data; drafting and editing of the manuscript or critical review of important intellectual content; intellectual participation in the propaedeutic and/or therapeutic conduct of studied cases; approval of the final version of the manuscript.

Renata Heck: Collection and acquisition of data; drafting and editing of the manuscript or critical review of important intellectual content; intellectual participation in the propaedeutic and/or therapeutic conduct of the studied cases; approval of the final version of the manuscript.

Maria Antonieta Rios Scherrer: Collection and acquisition of data; drafting and editing of the manuscript or critical review of important intellectual content; intellectual participation in the propaedeutic and/or therapeutic conduct of the studied cases; approval of the final version of the manuscript.

Luciana Paula Samorano: Collection and acquisition of data; drafting and editing of the manuscript or critical review of important intellectual content; intellectual participation in the propaedeutic and/or therapeutic conduct of the studied cases; approval of the final version of the manuscript.

Isabella Campos Machado Cordeiro: Collection and acquisition of data; drafting and editing of the manuscript or critical review of important intellectual content; intellectual participation in the propaedeutic and/or therapeutic conduct of the studied cases; approval of the final version of the manuscript.

Mariana de Figueiredo Silva Hafner: Collection and acquisition of data; drafting and editing of the manuscript or critical review of important intellectual content; intellectual participation in the propaedeutic and/or therapeutic conduct of the studied cases; approval of the final version of the manuscript.

Renata Marli Gonçalves Pires: Collection and acquisition of data; drafting and editing of the manuscript or critical review of important intellectual content; intellectual participation in the propaedeutic and/or therapeutic conduct of the studied cases; approval of the final version of the manuscript.

Lilith Sodré Eller: Collection and acquisition of data; drafting and editing of the manuscript or critical review of important intellectual content; intellectual participation in the propaedeutic and/or therapeutic conduct of the studied cases; approval of the final version of the manuscript.

Juliana Barros Rodrigues: Collection and acquisition of data; drafting and editing of the manuscript or critical review of important intellectual content; intellectual participation in the propaedeutic and/or therapeutic conduct of the studied cases; approval of the final version of the manuscript.

Ida Alzira Duarte: Design and planning of the study; drafting and editing of the manuscript or critical review of important intellectual content; approval of the final version of the manuscript.

Daniela Benzano Bumaguin: Design and planning of the study; collection of data, or analysis and interpretation of data; statistical analysis; approval of the final version of the manuscript.

Ruppert Ludwig Hahnstadt: Design and planning of the study; analysis and interpretation of data; approval of the final version of the manuscript.

## Conflicts of interest

None declared.
